# Computational Paradigm to Elucidate the Effects of Arts-Based Approaches: Art and Music Studies and Implications for Research and Therapy

**DOI:** 10.3389/fpsyg.2020.01200

**Published:** 2020-06-11

**Authors:** Billie Sandak, Avi Gilboa, David Harel

**Affiliations:** ^1^Department of Computer Science and Applied Mathematics, Faculty of Mathematics and Computer Science, The Weizmann Institute of Science, Rehovot, Israel; ^2^Department of Music, The Faculty of Humanities, Bar-Ilan University, Ramat-Gan, Israel

**Keywords:** computational technology, research method, artistic behavior dynamics, art, music, arts therapies, computer modeling, Computational Paradigm (CP)

## Abstract

Art therapy and music therapy, as well as other arts-based approaches and interventions, help to mitigate symptoms in serious and chronic diseases and to improve the well-being and quality of life for both healthy individuals and patients. Artistic creation is also researched and practiced intending to empower and understand individuals, groups, and communities. However, much research is required in order to learn how arts-based approaches operate and to enhance their effectivity. The complex and simultaneous occurrences involving the dynamics of the creation work, the client, and the therapist in a typical arts setting are difficult to grasp, consequently affecting their objective analyses. Here we employ our Computational Paradigm which enables the quantitative and rigorous tracking, analyzing, and documenting of the underlying dynamic processes, and describe its application in recent past and current real-world art and music studies with human participants. We aim to study emergent artistic behaviors of individuals and collectives in response to art and music making. Significant insights obtained include demographic variation factors such as gender and age, empirical behavioral patterns, and quantitative expressiveness and its change. We discuss the implications of the findings for therapy and research, such as causality for behavioral diversification and audio-visual cross-modality, and also offer directions for future applications and technology enhancements.

## Introduction

Arts-based approaches and interventions, such as arts therapies, are practiced to alleviate symptoms and induce psychosocial and therapeutic effects in a wide range of serious and chronic illnesses, conditions, and mental disorders, such as cancer, Parkinson, Alzheimer, physical disabilities, and schizophrenia. For example, pain, stress, depression, anxiety, fatigue, breathlessness, and other symptoms are mitigated by the use of *art therapy* (McNiff, 1992; Tusek et al., 1999; Nainis et al., 2006; Bar-Sela et al., 2007; Richardson et al., 2007; Thyme et al., 2009; Czamanski-Cohen et al., 2014), *music therapy* (Guzzetta, 1989; Pacchetti et al., 2000; Burns et al., 2001; Hilliard, 2003; Gold et al., 2004, 2009; Dileo, 2006; Dassa and Amir, 2014; Chang et al., 2015; Chen et al., 2016; Zhao et al., 2016; Hense and McFerran, 2017), dance/movement therapy (Sandel et al., 2005; Kiepe et al., 2012; Koch et al., 2014), drama therapy, and more (Graham et al., 2008; Rmunah, 2019). This is done for healthy individuals and for patients, in diverse age groups and populations, also enhancing one's well-being, quality of life, and ability to cope (Malchiodi, 2012; Skeja, 2014; Wang et al., 2014). Artistic creation is also researched and practiced with the intention of empowering and understanding individuals, groups, and communities (e.g., in music: Tuastad and Stige, 2015; Ansdell and Stige, 2016; and in art: Italia et al., 2008; Harrington, 2014; Huss and Sarid, 2014). The advantages of the engagement with the arts are also manifested in psychophysiological measurements, for example, in the reduction of heart rate, blood pressure, and cortisol levels (Kumar et al., 1999; Smolen et al., 2002; Lindblad et al., 2007; Chanda and Levitin, 2013; Batson et al., 2014; Belkofer et al., 2014).

Arts therapies have been employed clinically in hospitals, community centers, education facilities, clinics, and more for more than a century (Junge, 1994) and have been recognized as a profession for decades (Devlin, 2006; Misic et al., 2010; Wheeler, 2015). Arts-based approaches are carried out along the continuum of “arts as therapy” through “arts in therapy” (Bruscia, 1998; Dalley, 2009). In the latter notion, the therapist intervenes by trying to initiate changes, that is, connects and acts upon psychological dimensions of the arts experience, whereas in former, it is assumed that art or music making is the therapeutic process itself, and thus, the artwork or the musical work, for example, is the focus of attention. However, much research is required to unveil the underlying mechanisms by which such arts-based approaches operate and to improve their effectivity (Greenberg, 1994; Bell, 2002; Jones, 2005; Perruza and Kinsella, 2010; Stuckey and Nobel, 2010; McLean, 2014).

A three-way relationship forms a typical arts setting, that is, a setting utilizing the arts which consists of the creation work itself, i.e., the artwork or the musical work, the practitioner/therapist^1^, and the patient/client. All these entities constitute a dynamic environment, rich in complex occurrences and processes that are difficult to grasp. For example, in artwork, these include color and tool choice, the starting of a drawing stroke, its stopping, the direction of that stroke, its length and velocity, erasures, and pressure exerted on the drawing tools. In musical work, these include instrument choices, the beginning and end of a specific played musical note, its pitch, and intensity. In the arts-based session, social interaction also occurs between the therapist and the client, involving their verbal and non-verbal communication, such as body language, facial expressions, positions in the setting's space, and therapist intervention. These simultaneous, complex, and interwoven behavioral processes are considered intractable to human observers, say researchers and therapists, who usually also describe and summarize them verbally, consequently affecting their objective analyses and interpretation. Hence, unveiling the behavioral processes underlying arts-based approaches in action is required.

**Figure 1:**
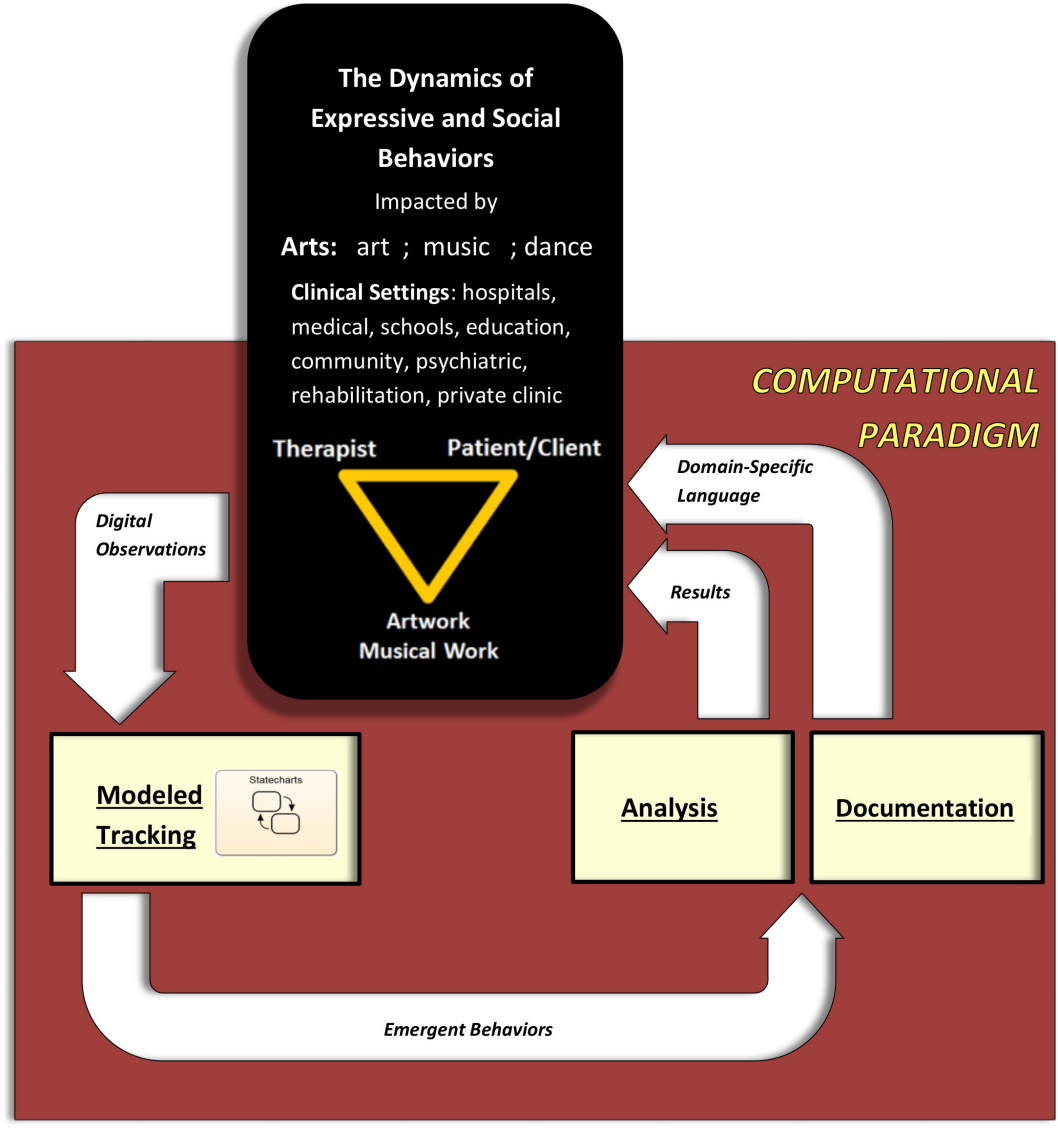
The Computational Paradigm (CP) design and its comprising components. Digital observations of the system under study, for example, artwork or music making, are fed into the **Modeled Tracking** module, which captures the occurring events, to yield emergent behaviors. These are input to the **Analysis** and **Documentation** modules, the first of which outputs empirical insights into the field of study, that is, art therapy or music therapy, and the second of which transforms the behavioral dynamics into an amenable description. Here, the application of the CP focuses on expressive behaviors for the art and music modalities. The rationale and additional details appear in the text.

**Figure 2:**
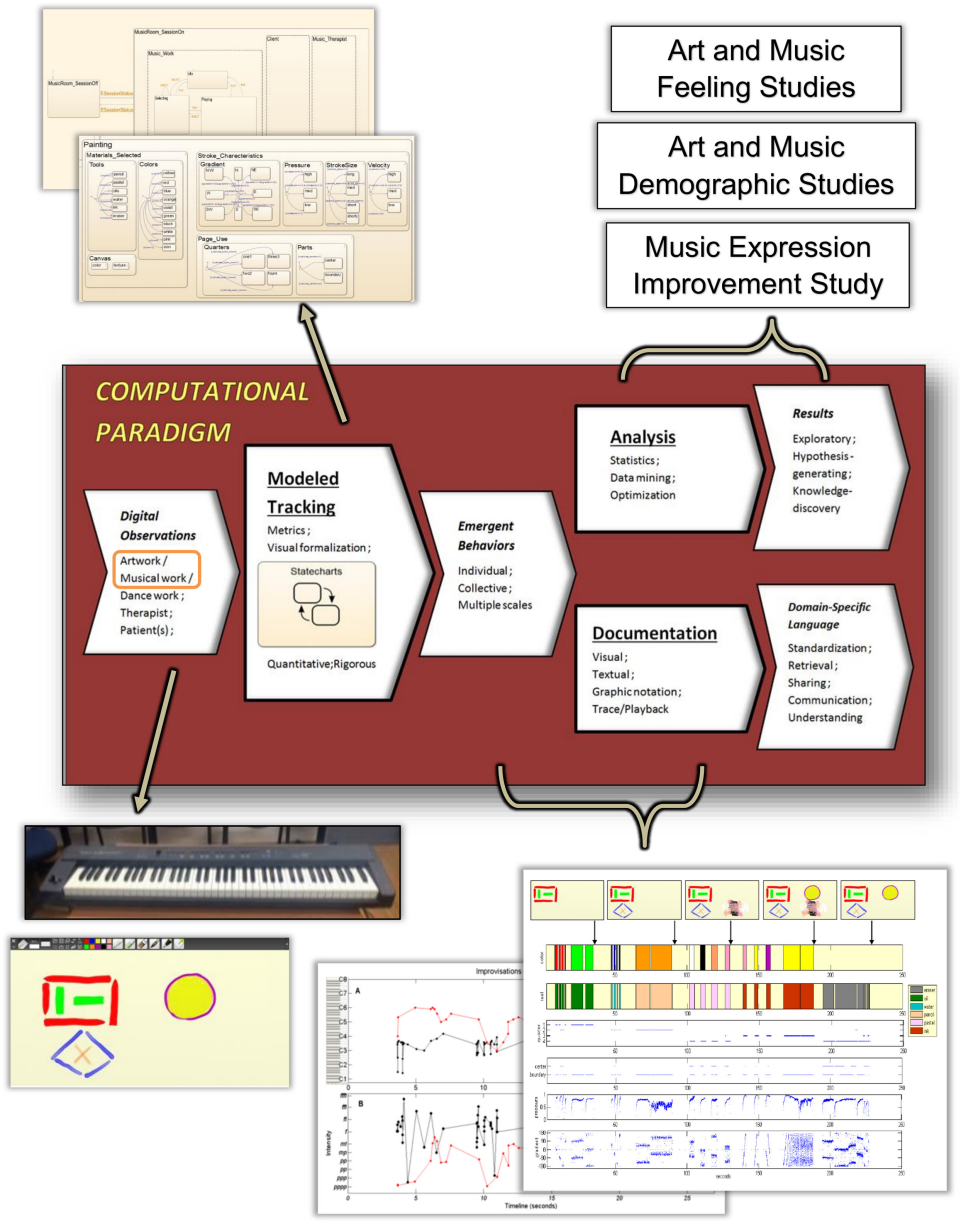
The CP applied to artwork and musical work. Tracking, analyzing, and documenting dynamic processes. See text.

We have designed a ***Computational Paradigm***(***CP***) (Sandak et al., 2015, 2019a,b) allowing the quantitative and rigorous tracking of expressive behavioral processes and their empirical analysis and documentation. See Figures 1, 2. This includes collecting data via digital inputs and defining and examining metrics and parameters for individual and collective performance analysis and comparison. All these allow mechanistic and systematic studies investigating the emergent behaviors (i.e., the arising properties and patterns of the dynamic processes) along the “arts as therapy” through “arts in therapy” continuum, to yield empirical discoveries. The rigorous tracking of the creation dynamics also allows us to discover important and significant phenomena that are otherwise missed when relying on the human eye of the observer or when examining only the end art product. Furthermore, this capability provides valuable information, that is, for evaluation and diagnosis of the client, for example, and for empirically monitoring the performance and progress of healthy and diseased clients.

The CP enables both intra-/local-/micro-analysis and inter-/global-/macro-analysis. The former relates to analysis where the focus is on specific moments within the dynamics of an arts-based session, whereas for the latter, to analysis carried out with reference to wider perspectives, for example, across sessions, individuals, and collectives (such as age and gender categorization). The technology enables carrying out empirically based, exploratory, hypotheses-generating, and hypotheses-testing investigations. All these are shown in this paper for the art and music modalities, focusing on the dynamic process of the creation work itself. Concentrating on the expressive behaviors via the artwork and musical work is the first step prior to the exploration of social behaviors via the therapist–client interaction, that is, focusing mainly on the left side of the “arts as therapy” through “arts in therapy” continuum. The CP, therefore, is applied here for studies that focus on art and music making and the expressive dynamics therein.

Using the CP, the demographic analysis of gender and age carried out for participants in response to several drawing tasks (Sandak et al., 2015) showed that females drew twice as fast as males; that males used the color blue and pencil for a time duration of about three times more than females did; that males used more types of drawing tools than females; and that older participants erased more than younger ones, while the latter used more colors. These results point to demographic variation factors in art drawing. This is hypothesized in art therapy sessions to be a cause for behavioral diversification originating from one's gender and age. The emergent musical behaviors we were able to discern from the study carried out in Sandak et al. (2019a) include the findings that males are more exploratory than females and that older participants express musical characterized negativity more than younger ones. That is, females employed a smaller range of notes and intensity values than males and even used fewer notes and pressed fewer keys. This is similar to a finding of the art modality research—namely, that females used fewer drawing tools than males. See also gender difference in spatial and exploration abilities (Brandner, 2007; Ellis et al., 2008). Age is also found to be a variation factor. The older subjects played with more keys pressed simultaneously and preferred the black keys, compared to the younger ones. That is, their style manifested the characteristics of negative valence. This is also reminiscent of the art research where older participants used fewer colors and erased more. Since gradual personality changes throughout life are well-known (Roberts and Mroczek, 2008; Harris et al., 2016; Kornadt et al., 2019), also in the musical life span perspective (Gembris, 2006; Gembris and Heye, 2014), we suggest researching a changed characteristic, that is, of artistic expressiveness. Providing therapists with these empirical findings can aid them with the information of demographic variation factors as one of the causes of behavioral diversification. That is, gender, age, and proficiency-level factors may be considered in treatment design and may help ameliorate its efficiency.

Here, we employ the technology for exploring artistic expression differences between participants asked to draw and play emotional themes, common procedures carried out in therapy sessions (Bruscia, 1987). These art and music feeling studies (*Study 1: Art Feeling Study* and *Study 2: Music Feeling Study*, respectively) yielded empirical behavior patterns and potential audio-visual cross-modality connections, that is, between art and music. We also apply the CP to real-world study in a music therapy setting aimed at improving one's musical expressiveness and quantifying it (*Study 3: Music Expression Improvement Study*). The implications of all of these findings for research and therapy are discussed, as well as possible future studies and technology developments.

Our epistemological stance is based on the notion that reality is complex and that occurrences are difficult to describe, and yet, that it is possible to find ways to describe reality and to find patterns and organizations of occurrences, sometimes even causal ones. We believe that events in therapy, as is true for all reality, are also extremely complex, and it takes much effort to describe a single clinical moment, let alone a whole session or a sequence of sessions. Nevertheless, it is the responsibility of therapists to understand events in treatment and to find patterns that might help and promote the client. We also think that researchers can develop tools that help therapists analyze and understand treatment, and that joint efforts of therapists and researchers promote the development of such tools. Finally, we believe that music therapy and art therapy, though focused on different modalities, have common bases when referring to clinical processes, relationships, emotions, creativity, and many other aspects.

## Methods

### Overview of the CP

Three major entities comprise the arts setting: the creation work (for example, the artwork or the musical work), the client, and the therapist. These components and their interactions constitute a dynamic system that continuously reacts to internal and external stimuli—what has been termed a *reactive system* (Harel and Pnueli, 1985). Within this system of the arts setting, the artwork and the musical work entities are specified as reactive sub-systems driven by stimuli of events. The art creation/construction work includes events such as picking up a drawing medium, e.g., a blue-colored pastel crayon or a paintbrush; starting to draw a stroke and stopping; exerting high pressure or low pressure on the drawing tool; or starting to erase. The musical work includes events such as choosing a musical instrument, say a piano keyboard; starting to play a specific musical note and stopping it; pressing the pedal of the piano; and choosing to play with the musical intensity of pianissimo (very soft), and then later with the intensity of mezzo forte (slightly loud) on a higher register (octave). These events actually transfer the system from state to state, for example, “materials being selected” to “painting,” or “instruments being selected” to “playing.” An “idle” state, for example, can be reached when the client is not active, for example, when he or she is busy thinking of the next drawing step or taking a rest, or when the therapist suggests that the client stop drawing/playing.

We employ the state/event approach for modeling the system, that is, the use of states and the events that trigger transitions between the states (described above)—an idea that has been used since the earliest days of describing computation. As depicted in Figures 1, 2, the CP suite consists of:

- The ***Modeled Tracking***module, responsible for capturing the dynamics of the system modeled, via digitized input. This allows the rigorous and objective capturing of the system's behavior, which is not possible by the naked human eye. We focus on the creation work as the first step in therapy investigations, prior to the exploration of the contribution of therapist–client interaction. Hence, the system studied is the artwork, input by tablet touchscreen, and the musical work, input by a digital piano keyboard (see bottom left side of Figure 2). This module hosts the system's model, which is *Statecharts* based (Harel, 1987; see top left side of Figure 2), and its defined art and music modality metrics/parameters. Statecharts is a visual formalism (Harel, 1988), which enriches the basic state/event modeling approach with means for describing hierarchy (nested states) and multi-level transitions, orthogonality (concurrent states), and more. We base the system modeling on Statecharts and use its underlying execution and analysis tools (MATLAB, 2015; Simulink, 2015; Stateflow, 2015) to track and analyze the artwork and the musical work systems.
- The ***Analysis***module, responsible for investigating the decoded emerging behaviors of individuals and collectives, in response to art making or music making. In this module, we use statistical, computational, and algorithmic tools to investigate the data output obtained by the Modeled Tracking module, as dictated by the studies' aims (see top right side of Figure 2 and the next sections describing Study 1 to Study 3).
- The ***Documentation***module, which transforms the expressive emergent behaviors into a format amenable to easy perception and contemplation. This is carried out by compiling textual and graphical reports to convey the properties of the dynamics of the artwork construction and music-making processes (see examples on the bottom right side of Figure 2 and the results).

### Art Modality Parameters

The CP allows: (i) **Time—**measuring exact time durations of occurrences within the art session, e.g., net drawing time and net idle time in which the client is not engaged in art activity. (ii) **Colors and Tools—**tracking time durations of erasure periods and the use of other drawing tools and color choices, as well as their switching frequency, preference profile, and cross-sections therein, for example, color palette per drawing tool. (iii) **Strokes—**capturing the characteristics of hand movements generating the strokes, e.g., smooth, sharp, jumpy, repetitive, and calculating their drawing velocity, direction, amplitude, and pattern, for example, circular (clockwise or counter-clockwise), as well as the total number of strokes generated, their accumulated length, and average size; also the pressure exerted on the drawing tools and erasure. (iv) **Page Canvas—**tracking the drawing page (canvas) area and time use during the art-making process, e.g., whether it was carried out in a confined area (say, a page corner or a particular section), on the page boundaries or in its center; also page crossing, that is, horizontal and vertical drawing movements across the page's sections.

### Music Modality Parameters

The CP enables: (i) **Time—**measuring and calculating exact time durations of occurrences within the music session, e.g., net playing time, net idle time in which the client is not engaged in musical activity or in pressing a key, and concurrent playing time, that is, playing time obtained from notes (keys) pressed in parallel. (ii) **Notes/Keys/Clusters—**tracking note use per time and per press, for example, net number of notes used, total number of notes pressed (a key can be pressed more than once), their time durations and density, and their cluster formations. *(*iii) **Intensity and Octave—**capturing and analyzing preference profile of octave use and note intensity, e.g., whether the music-making process is carried out in confined pitch values (registers) and intensity levels (musical dynamics). (iv) **Pitch Classes**—profiling pitch classes, that is, the overall note use distribution is collapsed onto an octave (C, C#, D,…, A#, B pitch), as well as chromatic preference (say, key color on a piano, that is, black/white). (v) **Transitions—**calculating transitions, e.g., diminuendo, crescendo, ritardando, accelerando, and chromatic (for example, white to black, black to black). (vi) **Pedal—**pedal use, such as number of presses and time durations.

Related work in the art domain (Kim, 2010) and the music domain (Luck et al., 2007; Streeter et al., 2012; Erkkilä et al., 2014) can be found in our publications (Sandak et al., 2015, 2019a,b), as well as a more detailed description of our technology and the Statecharts models of the system.

### The Current Studies

#### Study 1: Art Feeling Study

A dozen participants, half males and half females, were asked to express in drawing a negative feeling and a positive feeling, and to also draw an image of a house-tree-person. The participants had a mean age of 38.8 [standard error of mean (SEM) = 3.3], median of 35.5, and all were Israeli with an academic background in exact and natural sciences. They had no formal art training or professional painting experience; that is, they were laypersons. On a computer tablet, they had a choice of 6 drawing tools (oils, pastel, ink pen, water color, pencil, and an eraser) and a palette of 10 colors (white, yellow, orange, pink, skin, red, violet, green, blue, and black) for each of the tools.

#### Study 2: Music Feeling Study

The study involved 108 healthy/normal-hearing participants, half male and half female; with a mean age of 33.1 (SEM = 1.3), median of 28; all Israeli; faculty, administration, and campus students from the liberal and social science faculties, studying for their BA or MA degree. Half of the participants were professionals, that is, had playing experience or formal musical studies/training, and the other half were laypersons, i.e., either had some childhood playing training or had none. The participants were asked to improvise the notion of “ugly,” “beautiful,” “negative feeling,” and “positive feeling” on a MIDI (Musical Instrument Digital Interface) piano keyboard.

#### Study 3: Music Expression Improvement Study

We studied expressive emergent behaviors of four healthy clients (Subjects A–D) supervised by a music therapist, in a succession of six 50 min sessions. These are aimed at developing and increasing their expressivity via playing free improvisations and guided exercises on a piano keyboard. The subjects, 22–35 years old, had modest experience in improvisations, college-level musical education, and a few years of piano training, mostly during their childhood. Each session began and ended with a free improvisation, yielding 12 free improvisations for each subject. In between the starting and ending free improvisations, the subjects executed exercises and tasks by the therapist, playing either alone or accompanied by him.

## Results

### The Art Modality

#### Study 1: Art Feeling Study

**Figure 3:**
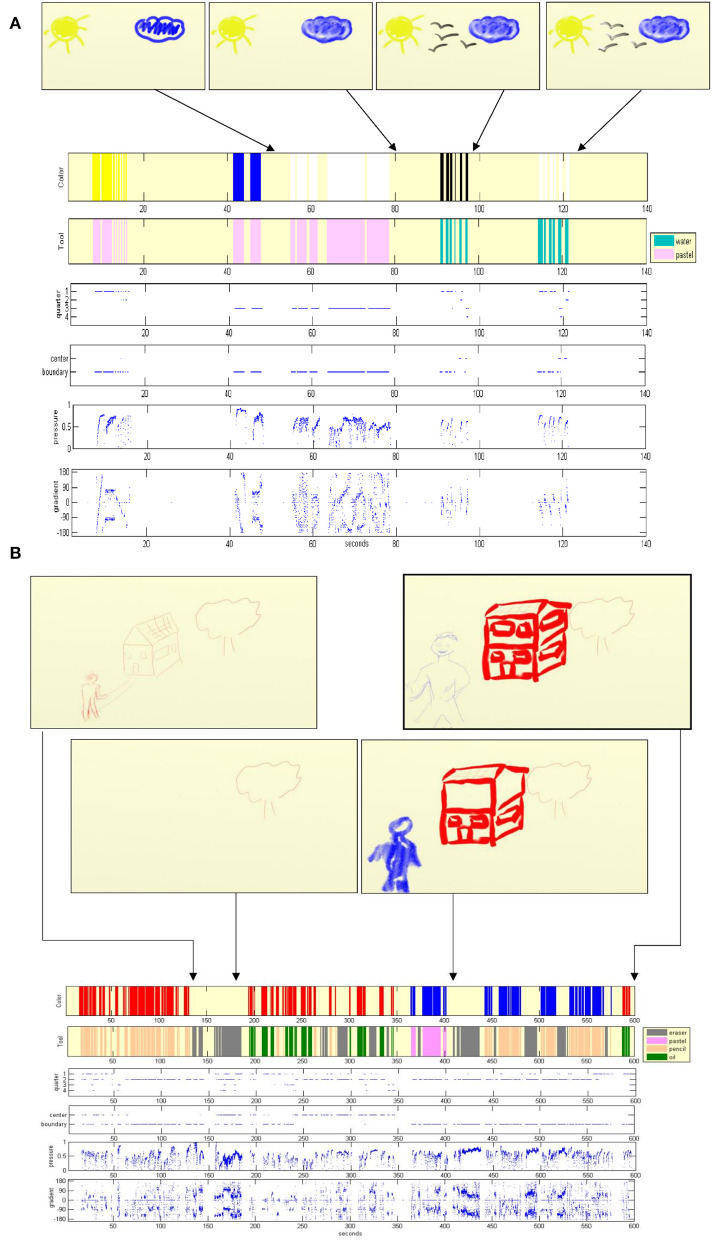
Visualization of art dynamics of individuals. A tracking graph of color and tool choices, erasures, time durations, their use and location on the drawing page, the pressure exerted on the tools, and drawing stroke gradient. **(A)** The visual report of an artwork imaging a positive feeling. **(B)** The artwork construction report of a house-tree-person.

We provide reports of the artwork construction, which enable the exploration and comparison of their dynamics. Figure 3 depicts the dynamics of art making of individuals. Therein, Figure 3A displays the visual report of the artwork imaging a positive feeling, which reveals the noticeable use of the color white (for 65% of the drawing time) in circular motions. The artwork construction of a house-tree-person in Figure 3B elucidates meaningful erasures (43% of the drawing time), where the house was changed twice and the person six times. In addition to the individual artwork investigations, we also conducted studies that involve collectives. These are described next.

**Figure 4:**
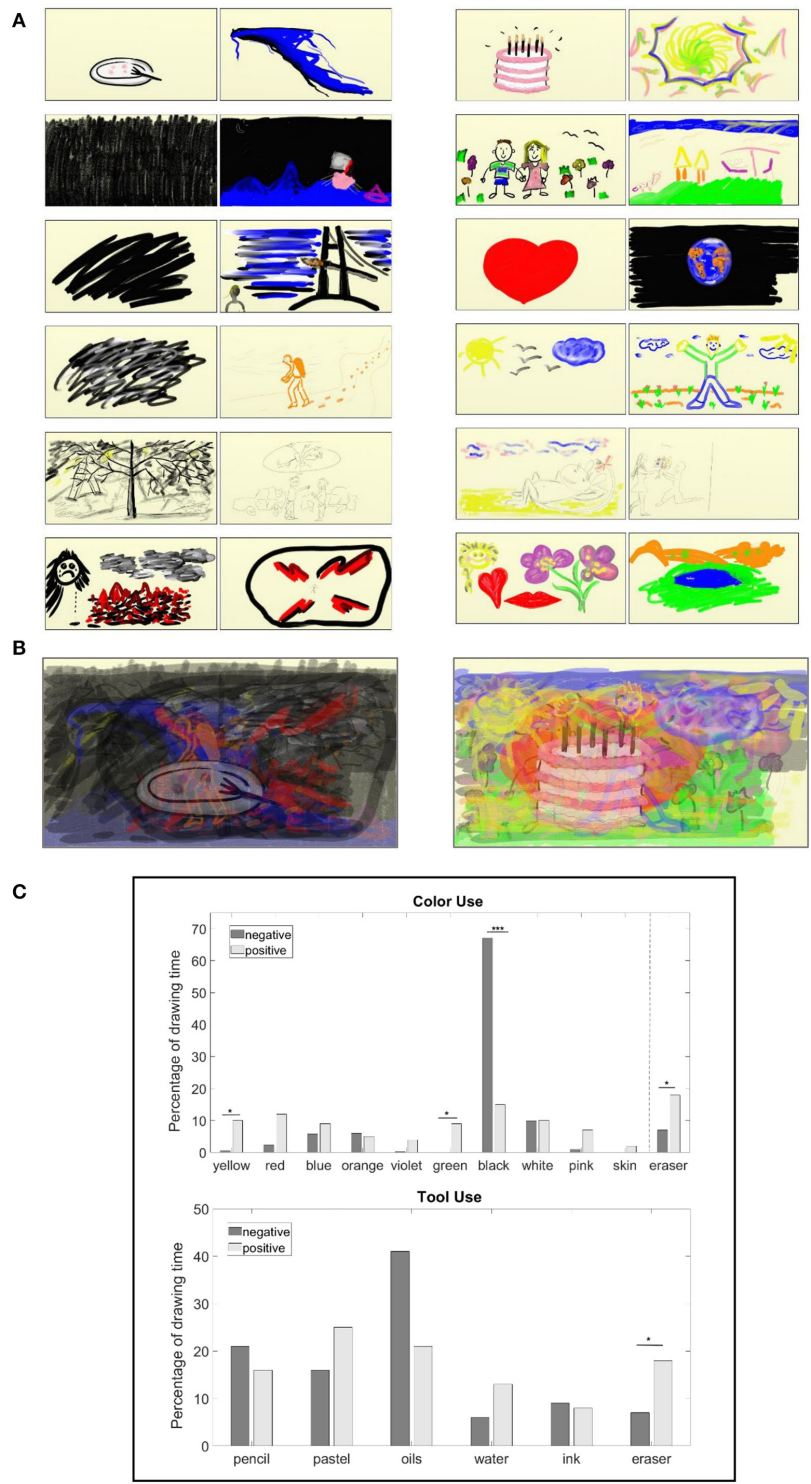
The participants' artworks imaging negative and positive feelings and their color and tool use. **(A)** The two left-hand columns correspond to the negative feeling artworks, whereas the two right-hand columns correspond to the positive feeling ones. **(B)** The left-hand panel is an artificial superposition of the negative feeling artworks, and the right-hand one is that of the positive feeling. The opacity for the superposition is 50%. **(C)** Comparison of the collective color and tool use as percentage of drawing time displayed in the upper and lower panels, respectively, for the negative feeling drawing task (dark gray) and positive feeling (light gray). *p < 0.05, ***p < 0.001. Additional parameter comparisons can be found in the table of Figure 5.

**Figure 5:**
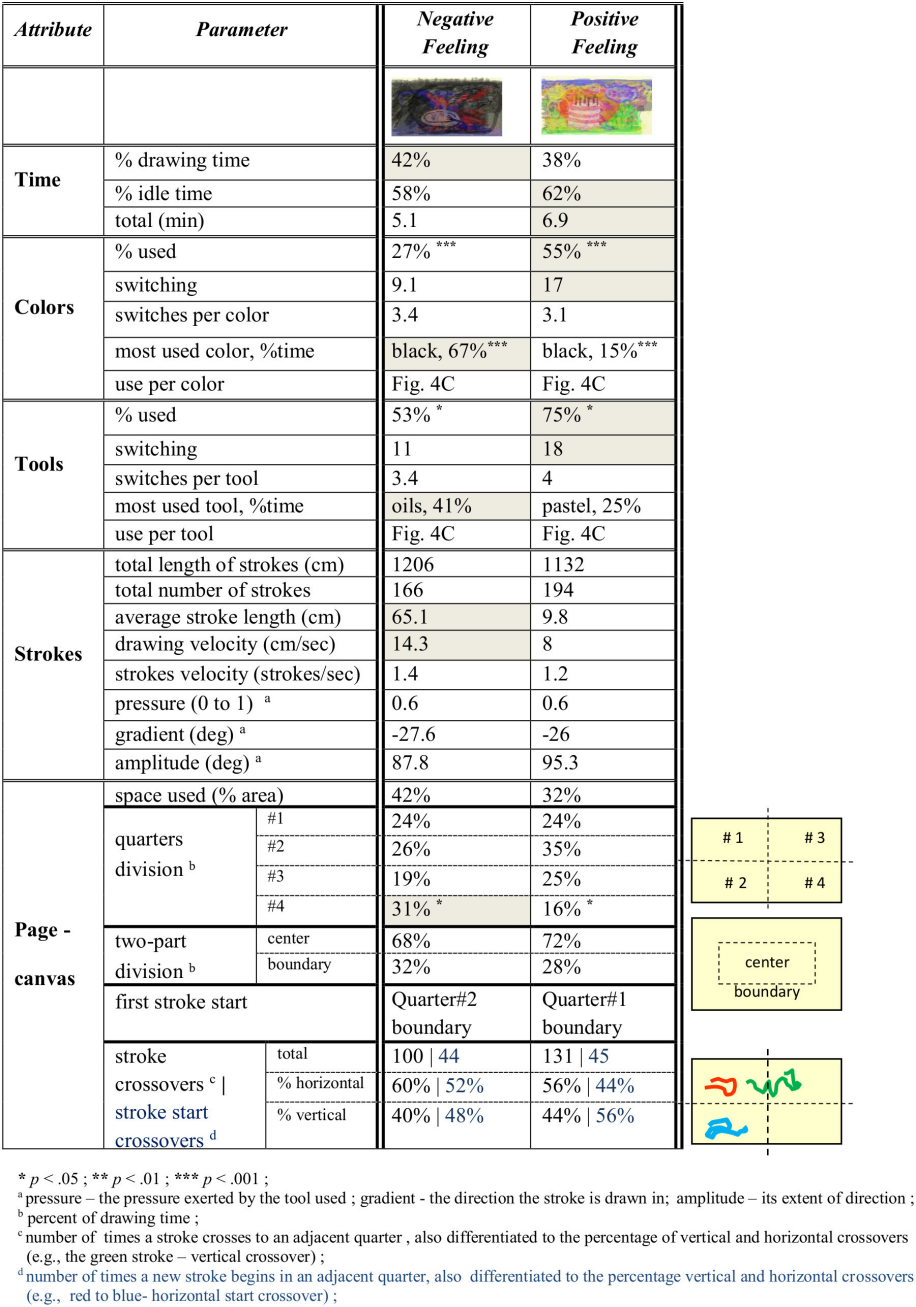
Parameter comparison of collective artwork construction processes. The processes compared are of the creation of negative and positive feeling images by participants displayed in Figure 4, and they report on the average parameter values for the performance of these task collectives.

We report on the two tasks of the participants drawing negative and positive feelings. See Figure 4A for the participants' artworks and Figure 4B for their respective collective superposition, emphasizing the color palette. The precise color and tool use in the negative and positive feeling artwork collectives can be seen in Figure 4C. The *F* statistic reported throughout this section is for *F*_(1, 23)_. The subscripts *n* and *p* identify the group task for the reported *SEM* values, that is, *n* for “negative feeling” and *p* for “positive feeling” (for example, *SEM*_*p*_)_._ We observe that, on average, the black color was used for 67% of the drawing time for the negative feeling drawings in comparison with 15% for the positive feeling (*F* = 28.15, *p* < 0.0001, η^2^ = 0.56, *SEM*_*n*_ = 8*, SEM*_*p*_ = 5.6). In addition, the oils were the most used tool for the negative feeling (41% of the drawing time), whereas they were used only 21% of the time for the positive feeling, in which pastel was preferable (25% of the drawing time). Furthermore, the negative feeling tasks were carried out with a drawing velocity of almost twice that of the positive feeling and with a higher percentage of net drawing time. Erasures were carried out significantly more in the positive feeling artworks, that is, 18% of the drawing time as compared with 7% of that of the negative feeling (*F* = 5.43, *p* = 0.03, η^2^ = 0.2, *SEM*_*n*_ = 2.6*, SEM*_*p*_ = 3.7*)*. Also significant is the use of yellow and green−10% of the drawing time for each of these in the positive feeling, as compared with 1% (*F* = *6.6, p* = 0.02, η^2^ = 0.23, *SEM*_*n*_ = 0.4*, SEM*_*p*_ = 3.5) and no use (*F* = 5.96, *p* = 0.02, η^2^ = 0.21, *SEM*_*n*_ = 0, *SEM*_*p*_ = 3.8) for the negative feeling, respectively. Red, pink, and skin colors were also preferred for the positive feeling. Further parameter comparisons appear in the table of Figure 5. Noticeable is the use of 55% of the colors and 75% of the tools for the positive feeling in comparison with 27% (*F* = 14.38, *p* = 0.0001, η^2^ = 0.4, *SEM*_*n*_ = 0.5*, SEM*_*p*_ = 0.56) use of colors and 53% of tools (*F* = 5.5, *p* = 0.03, η^2^ = 0.2, *SEM*_*n*_ = 0.4, *SEM*_*p*_ = 0.4) for the negative feeling, respectively.

### The Music Modality

The technology is also used for the investigations of individual and collective emergent behaviors in response to a number of free improvisation tasks (Study 2), as well as a music therapy study aimed at increasing one's expressivity following a succession of sessions with a music therapist (Study 3). These studies yielded interesting empirical discoveries, as we now describe.

#### Study 2: Music Feeling Study

The results reported for all participants, that is, for professionals and laypersons alike, appear in Sandak et al. (2019a), whereas in this work, we report on the group of lay participants alone. The improvisations carried out by professionals and laypersons expressing “negative feeling” and “ugly” were quantitatively significantly different from those expressing “positive feeling” and “beautiful.” The negative valence involved the use of lower-pitch notes, higher intensity values, the pressing of a cluster of keys, and dissonance playing. The notion of “ugly” required the use of even more keys than “negative feeling,” while “beautiful” involved less intensity than “positive feeling.”

**Figure 6:**
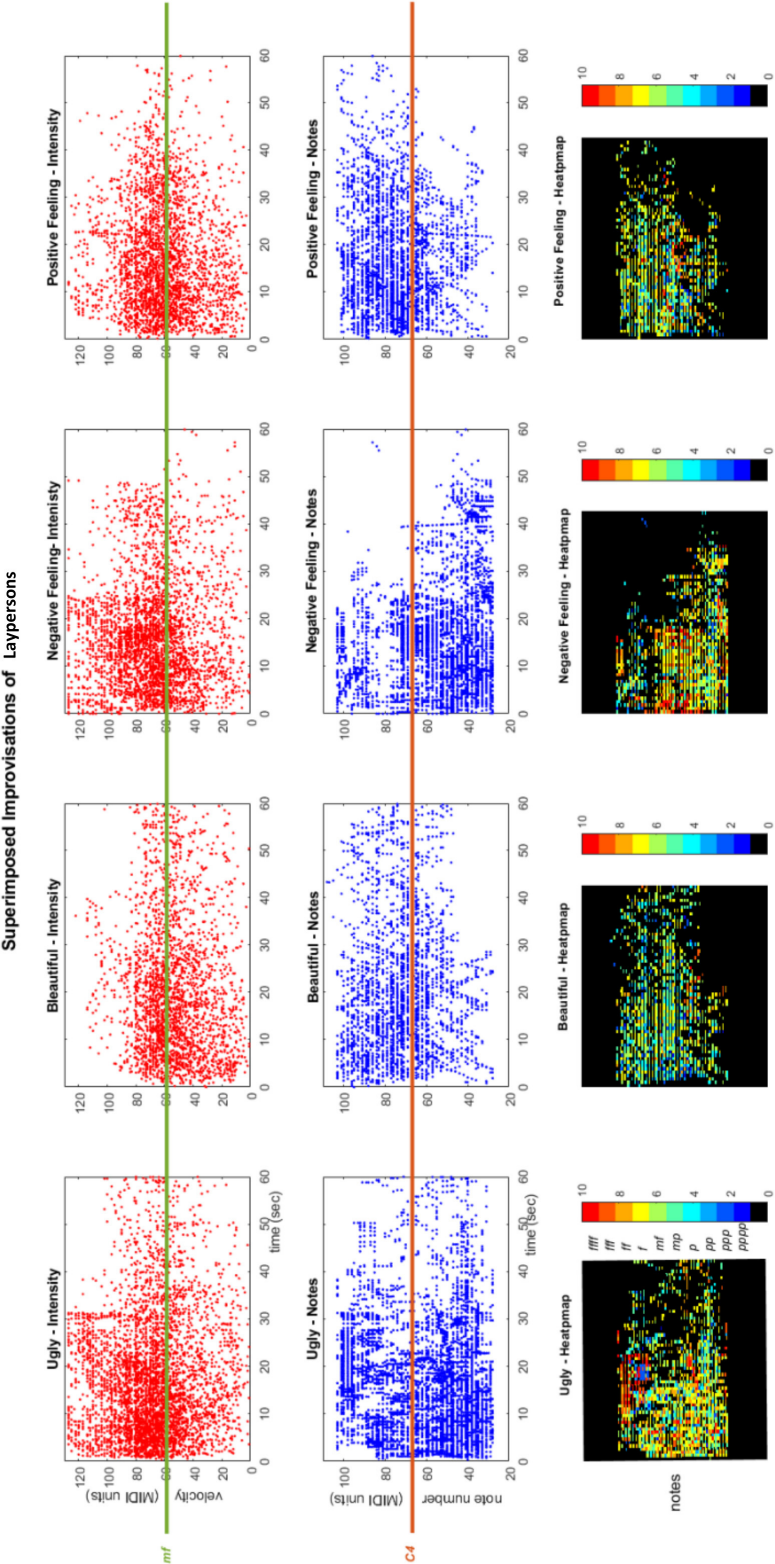
The participants' musical works improvising the titles of ugly, beautiful, negative, and positive feelings. The graphs depict these improvisations superimposed, where the x-axis displays their timeline. **(Top Row of Panels)** The intensity of the superimposed improvisations over time. The y-axis shows the notes' intensity values in MIDI (Musical Instrument Digital Interface) units (*pppp* to *ffff* discretized from 0 to 127), and the green line marks the *mf* intensity (mezzo forte—slightly loud) for visual comparison. **(Middle Row of Panels)** The notes pressed of the superimposed improvisations over time. The y-axis shows the note values in MIDI units (notes are numbered from 0 to 127), and the orange line marks the *C4* note (the middle C—C in octave no. 4) for visual comparison. **(Bottom Row of Panels)** Heatmap of the notes pressed and their intensity values (in color) of the superimposed improvisations over time. The y-axis shows the note values in MIDI units as appearing on the above row of panels, where their color display corresponds to the intensity values of these notes, ranging from *pppp* to *ffff* (see the colormap).

We now report on the group of lay participants alone. See Figure 6 for the graphical depiction of the superimposed results of laypersons improvising the notions of “ugly,” “beautiful,” “negative feeling,” and “positive feeling.” As for all participants, it is seen in the top row of panels and in the bottom row of heatmap panels of Figure 6 that the “beautiful” and “positive feeling” superimposed improvisations are “colder” than those of “ugly” and “negative feeling.” That is, the keys were pressed with less intensity, whereas “positive feeling” is a bit “warmer” than “beautiful,” owing to the more jolly/happy music played. The distribution of the superimposed notes pressed by laypersons appearing in the middle and bottom rows of panels in Figure 6 is similar to that obtained for all participants, laypersons and professionals alike, for example, higher-pitch notes in the “positive feeling” improvisations.

**Figure 7:**
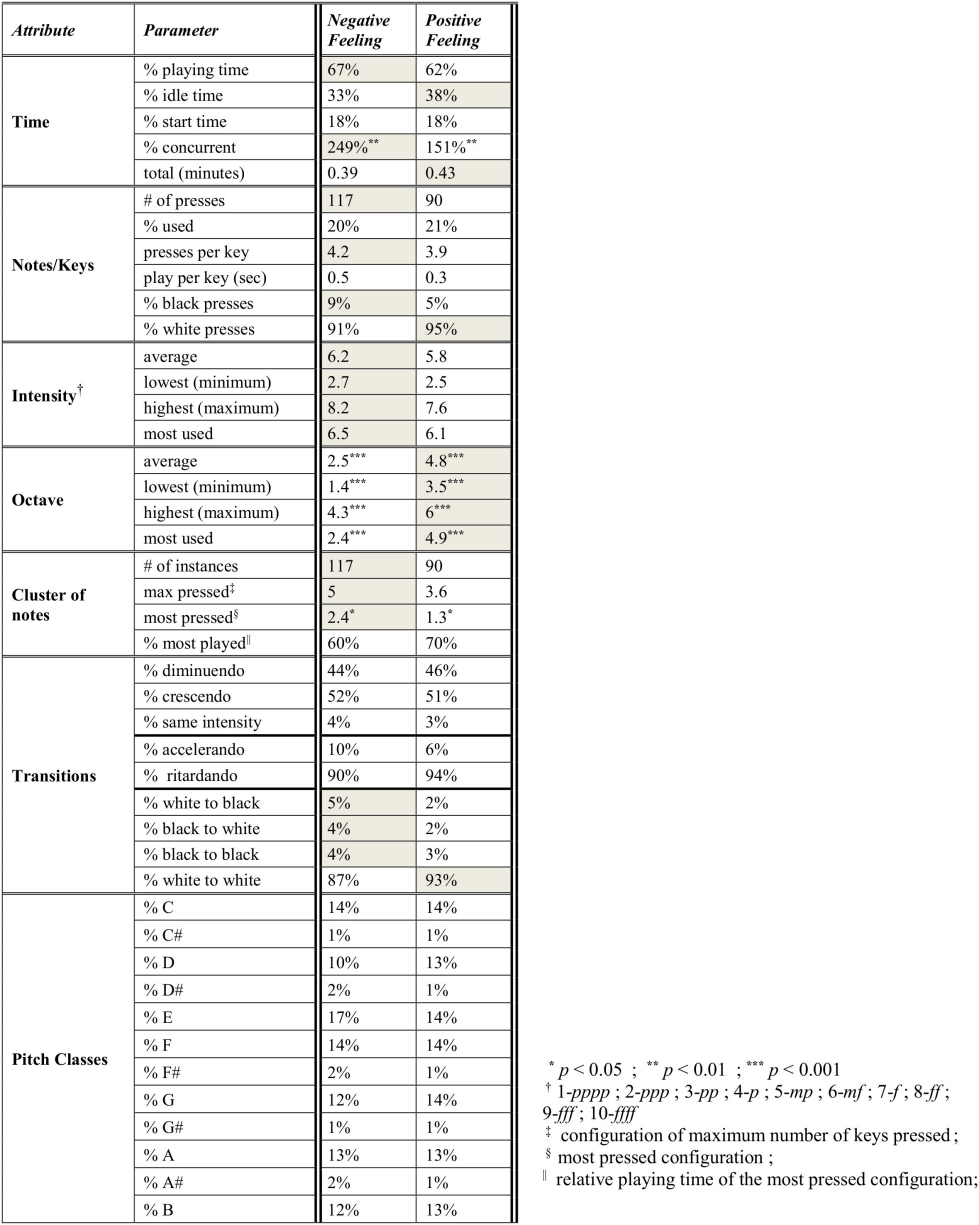
Parameter comparison in music improvisation making of collectives. These represent the averaged parameter values of improvised titles of negative and positive feeling by the participants (depicted as superimposed improvisations in the two right columns of the panels in Figure 6, and they can be heard as such by playing the Audios S1, S2, respectively).

In the table of Figure 7, we display the precise results of the “negative feeling” and “positive feeling” tasks of this group of layperson subjects. The *F* statistic reported throughout this study is for *F*_(3, 215)_. The subscripts *n* and *p* identify the group task for the reported mean (*M*) and *SEM* values, that is, *n* for “negative feeling” and *p* for “positive feeling” (e.g., *Mn* and *SEMp*). As displayed in the table of Figure 7, the comparison of octave use for these tasks resulted with significant mean differences between the average lowest, highest, and most used octave with respective values of *F* = 62.8, *p* < 0.0001, η^2^ = 0.47, *Mn* = 2.5, *SEMn* = 0.12, *Mp* = 4.8, *SEMp* = 0.18; *F* = 52.8, *p* < *0*.0001, η^2^ = 0.43, *Mn* = 1.4, *SEMn* = 0.26, *Mp* = 3.5, *SEMp* = 0.13; *F* = 14.38, *p* < 0.0001, η^2^ = 0.17, *Mn* = 4.3, *SEMn* = 0.17, *Mp* = 6, *SEMp* = 0.13; and *F* = 58.5, *p* < 0.0001, η^2^ = 0.45, *Mn* = 2.4, *SEMn* = 0.2, *Mp* = 4.9, *SEMp* = 0.14. That is, the “negative feeling” improvisations were played on lower octaves than those of “positive feeling.” The “negative feeling” improvisations also resulted in stronger-pressed keys, that is, notes with higher intensity than those of “positive feeling.” See the keyboard use also in the two right columns of panels in Figure 6. The “negative feeling” also resulted in a higher concurrent playing metric (that is, keys pressed together, for example, two keys pressed in parallel throughout the session play time yields 200% concurrent playing percentage), which took up 249% of the net playing time, in comparison with the 151% of “positive feeling” (*F* = 11.23, *p* = 0.01, η^2^ = 0.14, *SEMn* = 33, *SEMp* = 7). That is, larger clusters of keys were played in parallel in the “negative feeling” improvisations, as also seen for the metrics of maximum and most-pressed cluster of notes in the table of Figure 7, as well as preference for the black keys and the key transitions involving these.

#### Study 3: Music Expression Improvement Study

**Figure 8:**
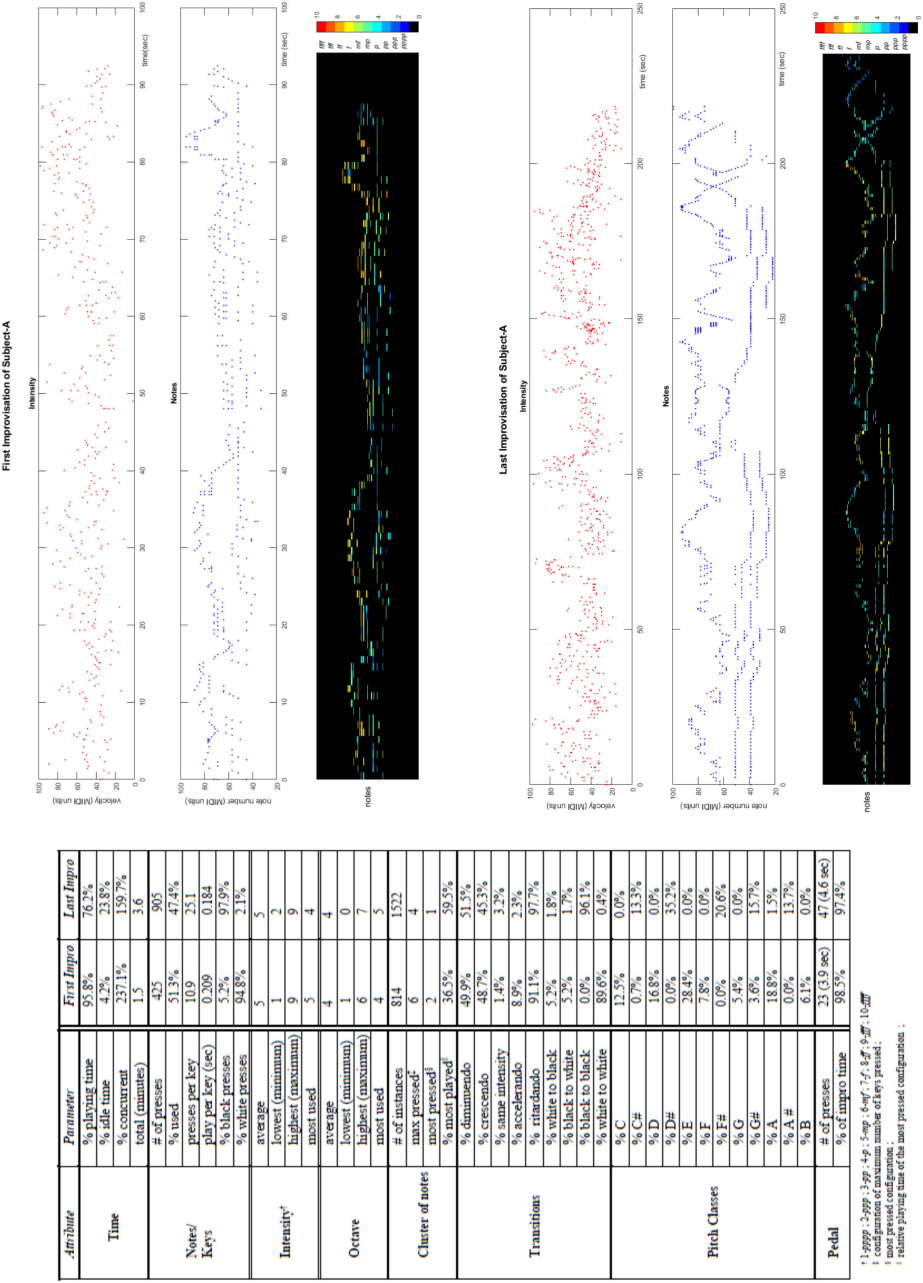
Parameter comparison and visualization of musical works of an individual. The first and last improvisations of Subject A, extracted from the first and sixth sessions in which the subject participated, respectively. **(Table)** The parameter comparison of these two improvisations. **(Top Right 3 Panels)** The timeline of the first improvisation, showing the intensity of notes, the notes themselves, and the heatmap of the notes as a function of their intensity (in color). The y-axis is in MIDI units. The colormap of the intensity values ranging from *pppp* to *ffff* is on the right. **(Bottom Right 3 Panels)** The timeline of the last improvisation.

**Figure 9:**
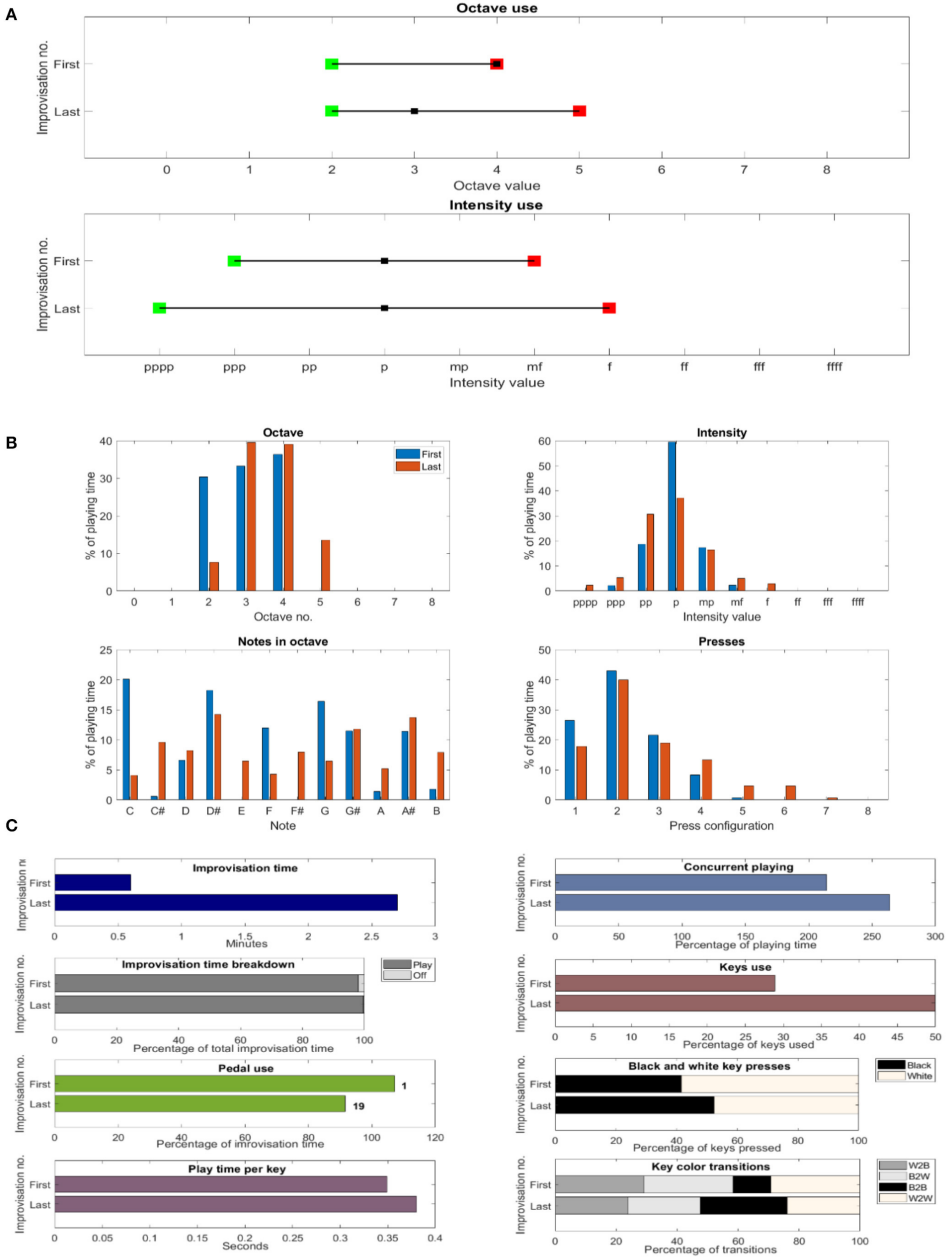
Comparable empirical depiction of musical works of an individual. The first and last improvisations of Subject B, extracted from the first and sixth sessions, respectively. **(A)** Range of octave and intensity use. **(B)** Distribution of the percentage of playing time for octave and intensity use, pitch classes and note clusters. **(C)** Additional comparisons of music parameters.

We applied the CP to real-world study in a music therapy setting. This subsection is based on the adaptation of a mathematical conference paper (Sandak et al., 2019b) and the introduction of new findings as well. The results obtained from analyzing the first and last improvisations exhibit improved expressiveness, whereas the empirical measurements allow the rigorous comparison of the performances and quantifying the change in one's expressivity. See Figures 8, 9 for Subject A and Subject B results, respectively, as discussed next.

Subject A's results are presented in the table and plots of Figure 8. The improvisation duration almost doubled from 1.5 min for the first improvisation to 3.6 min for the last, whereas the pedal's average use grew from 3.9 s per press to 4.6 s. Noteworthy are the black key presses in the last improvisation as compared to the first (almost all white), their transitions (see “% black to black”), and the pitch class distribution. This capability adds important and accurate information to the therapist's summary, including, for example, the use of the “white” note A. The actual improvisation evolution along its timeline is depicted in the panels on the right side of Figure 8, where the notes pressed and their intensity are merged onto a heatmap display. In the first improvisation, the right hand of Subject A pressed with more intensity in comparison to the left hand (hotter). During the last improvisation, the intensity evened out for both hands. Also clearly displayed are the chords played by the left hand there.

As seen in the top panel of Figure 9A, the range of Subject B's octave use grew. That is, for the first improvisation, the second octave was the minimum octave used (marked by a green square), and the fourth was the maximum one (in red), which changed to the higher fifth octave in the last improvisation. The most used octave changed from the fourth octave to the third (black square), whereas the histogram appearing in the top left panel of Figure 9B depicts the exact distribution of the octave number use per percentage of playing time. As seen in the bottom panel in Figure 9A, the range of intensity values (dynamics) also grew, whereas the top right panel of Figure 9B displays the intensity values histogram. Noteworthy is the increased use of the black keys, i.e., the key color distribution of presses and their transitions, as appearing in Figure 9C's two bottom right panels, respectively, overall tending to more chromaticity as seen in the pitch class preference of the bottom left panel of Figure 9B. A meaningful change is also seen in the top left panel of Figure 9C, depicting the total time duration of the improvisation length, which increased from 0.6 min of the first to more than four times in length, that is, to 2.7 min. In fact, all four subjects increased the durations of their improvisations, probably more comfortable in expressing themselves. The pedal was used statically by Subject B, placing her foot on it in the first improvisation; however, during the last improvisation, free use was captured (Figure 9C, third left panel). Owing to the larger clusters of key presses (Figure 9B, bottom right panel), as well as almost “doubling” the percentage of key use, from 29 to 50% (Figure 9C, top right second panel), notable is the increase in the concurrent playing time (Figure 9C, top right panel). An additional outcome of the detailed and precise technology is to compare it with the therapist's written summary. He described the first improvisation as “a short improvisation,” and the last one was not documented at all.

We found and provide empirical evidence of change, for example, indication of: (i) the ability to express more varied emotional states via enhanced intensity range; (ii) indication that the client has the ability to use more notes to express him/herself through enhanced octave use; (iii) more opportunities to express feelings and situations via chromatic key transitions, as usually, clients tend to “adopt” white keys or black keys or to avoid chromaticity at first; (iv) an improvement in expressive abilities by frequent pedal use that enables more shades of expression; and (v) broader expressive possibilities through enhanced concurrent note use that requires playing with more than one finger at a time.

## Discussion

### Summary and Implications

**Figure 10:**
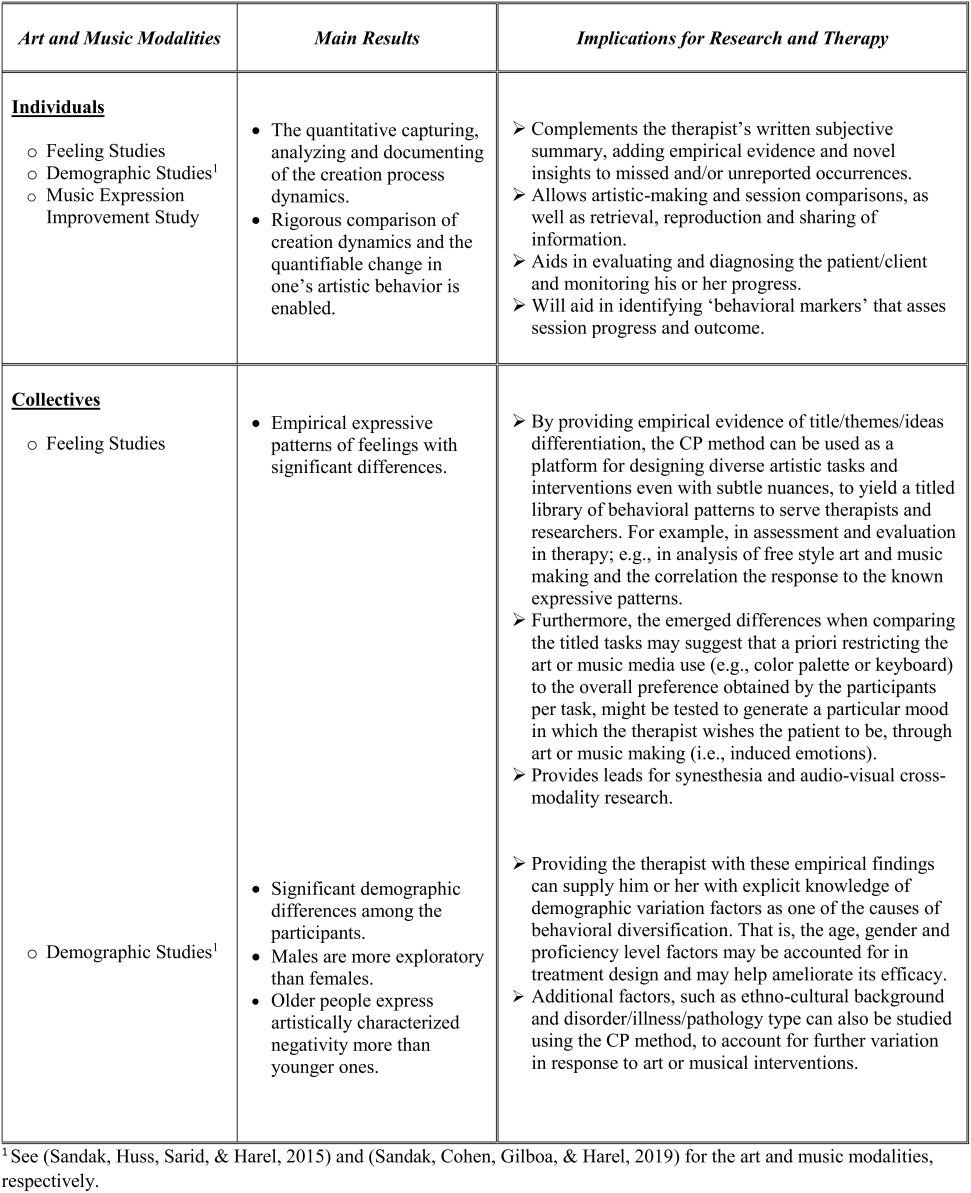
Summary of the CP utilization and contribution to research and therapy. Further details can be found in the text and in the references (Sandak et al., 2015, 2019a,b).

We have shown that the technology is useful in providing rigorous and significant insights and empirical probing abilities for research and therapy. The results and implications of the use of the CP for practice, research, and theory in this work, and in previous work (Sandak et al., 2015, 2019a,b), are summarized in the table of Figure 10 and discussed here.

The technology accurately captures, analyzes, and documents the dynamics of emergent behaviors and change and thus may: (i) complement the therapist's written summary, adding empirical evidence to unreported and/or missed occurrences; (ii) allow the comparison of improvisations and sessions, as well as sharing information between experts; (iii) facilitate evaluating and diagnosing the client and progress; and (iv) assess the quality of the professional work of the therapist in comparison to others.

Emerging behavioral properties generated by the CP include demographic variation factors that may point to a cause for artistic diversification, that is, artistic behavior that may be dependent on gender, age, and professional level. Furthermore, empirical behavior patterns characterize expressive emotional themes and thus may facilitate identifying or inducing moods and/or attitudes. The observed potential connections between art and music may contribute to the research of audio-visual cross-modality. We now discuss this further.

There are several clinical implications to the art feeling study (Study 1). First, the empirical differentiation and comparison of tasks can be utilized in creating a library of titled emotions/feelings decoded as empirical behavioral patterns. For example, this library can facilitate identifying the client's emotional state in freestyle drawing by comparing its dynamics to the stored patterns there. Second, we also suggest that the colors' and tools' significant preference may lead to studies which have to do with art-induced emotions. For example, we postulate that limiting the palette to green and yellow hues (as well as shades of reds), and the tools to pastel and erasure, may induce a positive feeling via drawing. As with the art study, such results of the music feeling study (Study 2) can lead to a titled library of empirical behavioral patterns to serve researchers and therapists, for example, in analyzing freestyle improvisations and correlating them to the known expressive patterns. Furthermore, we have postulated that a priori restricting the keyboard use to the preferences exhibited by the participants per task might be tested for induced emotions, that is, to explore, through playing, a particular mood or state of mind which is relevant at that point in therapy^2^ (Nakahara et al., 2011; Vuoskosky and Eerola, 2012; Menninghaus et al., 2017).

An additional phenomenon to research is that of *synesthesia*, a bioneurological phenomenon where stimulation of one sensory/cognitive modality pathway leads to automatic and involuntary experiences in a second one, for example, the audio-visual cross-modalities, as also is being experienced by non-synesthetes (Sagiv and Ward, 2006). The precise analysis of musical data of Study 2, compared to the results of Study 1, which focused on visual data, can be the basis for a systematic exploration of synesthesia, as these utilize universal mechanisms, and their mapping “deserves systematic attention” (Sagiv et al., 2009). That is, we aim to map the parameters of auditory and visual image creation, in the search for emergent attributes connecting them and potential synesthetic connections. Both synesthetes and non-synesthetes tend to choose brighter colors with higher-pitch sounds (Hubbard and Ramachandran, 2005), as has also been shown here, and also obtain the specific color hues that manifest this, such as yellow and green. As seen in the tables in Figures 5, 7, one might hypothesize that high pressure exerted on the drawing tools may be connected with higher intensity exerted on the piano keys. However, no differences in pressure appeared between “negative” and “positive” drawings. Nevertheless, in the “negative” valence, for example, the significant use of oils may be mapped to high intensity, high drawing velocity can be mapped to large clusters of notes pressed, and black color can be mapped to low-pitch sounds. These potential leads should be further studied.

The technology can be used by the scientific and clinical communities as follows: (a) The empirical findings of emerging behavioral properties we obtain can be used by therapists and researchers as new or complementary data in their own work. (b) In the long run, it may be used as a research method by other workers for other empirical-based investigations. (c) These may also lead to additional research avenues, in which the behavioral results we generate (such as age and gender difference in the processes of art and music making) are mapped onto bio-neural mechanisms, for example, brain activity (Raglio et al., 2006; Bolwerk et al., 2014).

Eventually, we expect our ideas to be used also in assessing and predicting treatment progress and outcome, with the intention of improving and optimizing it too. We now expand upon this.

### Future Goals

#### Modeled Tracking Module

The CP is designed to capture and track additional musical instruments other than the one we used here (a MIDI piano keyboard). This capability will be employed in investigations where the client's choice of instrument is part of the research, and hence, we plan to include MIDI-based woodwind, percussion, and string instruments. We also plan to expand the technology to accommodate real art materials as well as acoustic instruments, and expect to further implement the CP to capture occurrences in 3D and audio space. As such, sculpting may also be studied. These data too will serve as input to the models of client and therapist that we plan to develop. The models will track bodily/non-verbal and auditory/verbal dynamics that capture social interaction, such as body language, facial expression, and therapist intervention. This will also enable model development for the dance/movement modality. Initial steps in all these directions can be found in Sandak et al. (2015, 2019a).

#### Analysis Module

The Analysis module, which is study-dependent, analyzes the dynamics of the emergent behaviors provided by the Modeled Tracking module (see Figures 1, 2). It will enable the following: (i) We will determine which of the parameters (as in the tables in Figures 5, 7) evaluate and assess the session progress and outcome. That is, we shall identify “behavioral markers,” such as those portraying change. We may thus be able to reveal significant markers (via data mining, for example) that are universal, or at least common to many clients or to some specific groups thereof, such as age group, gender, and illness. This is also true for the quantitative identification of “turning points” or “moments of change” and their causality (Greenberg, 1994; Bell, 2002; Holmqvist et al., 2017). (ii) The demographic investigations described earlier constitute one phase in a broader investigation for discovering additional differences in the dynamics of image and auditory creation. Factors such as ethno-cultural characteristics, illness, and disorder type may account for individual and collective variation in response to arts intervention. For example, it would be interesting to study minority ethnic groups (e.g., non-Western) for their image creation mechanisms portraying internal feelings, such as their sense of well-being, affiliation, coherence, and civic engagement. (iii) We also wish to analyze the musical parameters for their syntax, for example, structure and harmony, and art drawing for shapes, sizes, level of figurativeness, and abstractedness. (iv) Eventually, we also aim to optimize arts-based interventions, that is, to fit the choice of specific art modality according to the client's characteristic factors, such as illness or demographics.

#### Documentation Module

We have enabled the empirical reporting of artistic behavioral patterns, hoping to devise an appropriate formal language for depicting the dynamics of the arts-based session. This will allow session comparison and documentation, as well as the retrieval and sharing of information. Preliminary graphical notation exemplification for music therapy sessions can be found in Figures 8, 9, and in Bergstrom-Nielsen (2009) and Gilboa and Bensimon (2007). We plan to further develop these, as well as our textual and visual reports of the dynamics of art and music making, to yield automated or semi-automated *domain-specific languages*. We also plan to use these in real time throughout the session, as a “dashboard” for the therapist. Once a formal language is agreed upon and adopted in a particular domain of activity, it will ease the communication and understanding between specialists and communities of the domain's fields.

We thus believe that the CP may facilitate making significant progress in scientific and clinical arenas that employ the arts, such as psychology, healthcare, social work, education, and recreation.

## Data Availability Statement

The datasets generated for these studies are available on request to the corresponding author.

## Ethics Statement

The studies involving human participants were reviewed and approved by The Weizmann Institute's Bioethics and Embryonic Stem Cell research Oversight (ESCRO) Committee and the Bar-Ilan University's Ethics Committee. The patients/participants provided their written informed consent to participate in this study.

## Author Contributions

BS, AG, and DH: conceptualization, formal analysis and writing—review and editing. BS: data curation and writing—original draft.

## Conflict of Interest

The authors declare that the research was conducted in the absence of any commercial or financial relationships that could be construed as a potential conflict of interest.

## Footnotes

^1^The terms practitioner and therapist are interchangeable as are the terms patient and client. We will use therapist and client throughout.

^2^See Priestley (1975) for an example of a model that works with a variety of clinical techniques designed to explore feelings and emotional states through improvisation.

## Supplementary Material

Audio S1 Superimposed improvisations of negative feeling (MP3).

Audio S2 Superimposed improvisations of positive feeling (MP3).
